# The effects of exercise on cognition in Parkinson’s disease: a systematic review

**DOI:** 10.1186/2047-9158-3-5

**Published:** 2014-02-24

**Authors:** Danielle K Murray, Matthew A Sacheli, Janice J Eng, A Jon Stoessl

**Affiliations:** 1 Pacific Parkinson’s Research Centre and Department of Medicine, Division of Neurology, University of British Columbia & Vancouver Coastal Health, Vancouver, BC V6T 2B5, Canada; 2 Department of Physical Therapy, University of British Columbia, Vancouver, BC V6T 1Z3, Canada

**Keywords:** Parkinson’s disease, Exercise, Cognition, Humans, Animals

## Abstract

Cognitive impairments are highly prevalent in Parkinson’s disease (PD) and can substantially affect a patient’s quality of life. These impairments remain difficult to manage with current clinical therapies, but exercise has been identified as a possible treatment. The objective of this systematic review was to accumulate and analyze evidence for the effects of exercise on cognition in both animal models of PD and human disease. This systematic review was conducted according to the Preferred Reporting Items for Systematic reviews and Meta-Analyses (PRISMA) statement. Fourteen original reports were identified, including six pre-clinical animal studies and eight human clinical studies. These studies used various exercise interventions and evaluated many different outcome measures; therefore, only a qualitative synthesis was performed. The evidence from animal studies supports the role of exercise to improve cognition in humans through the promotion of neuronal proliferation, neuroprotection and neurogenesis. These findings warrant more research to determine what roles these neural mechanisms play in clinical populations. The reports on cognitive changes in clinical studies demonstrate that a range of exercise programs can improve cognition in humans. While each clinical study demonstrated improvements in a marker of cognition, there were limitations in each study, including non-randomized designs and risk of bias. The Grading of Recommendations Assessment, Development and Evaluation (GRADE) system was used and the quality of the evidence for human studies were rated from “low” to “moderate” and the strength of the recommendations were rated from “weak” to “strong”. Studies that assessed executive function, compared to general cognitive abilities, received a higher GRADE rating. Overall, this systematic review found that in animal models exercise results in behavioral and corresponding neurobiological changes in the basal ganglia related to cognition. The clinical studies showed that various types of exercise, including aerobic, resistance and dance can improve cognitive function, although the optimal type, amount, mechanisms, and duration of exercise are unclear. With growing support for exercise to improve not only motor symptoms, but also cognitive impairments in PD, health care providers and policy makers should recommend exercise as part of routine management and neurorehabilitation for this disorder.

## Introduction

### Rationale and objective

Aside from well-documented motor symptoms, most Parkinson’s disease (PD) patients suffer from associated non-motor complications, including cognitive impairment, mood disorders, olfactory dysfunction, sleep disturbance, fatigue and anxiety [1-3]. Of the non-motor symptoms, cognitive impairments are particularly prevalent in PD with up to 83% of patients developing dementia after 20 years [4]. The non-motor symptoms of PD can be at least as detrimental as motor manifestations for a patient’s health and overall quality of life, but unfortunately remain difficult to manage with current clinical therapies [1-3].

The current gold-standard for testing global cognitive capacity in clinical practice includes objective verbal and written tests. Of the quick screening cognitive tests available, the Montreal Cognitive Assessment (MoCA) [5] has been widely accepted for use in PD populations [6] by assessing multiple domains of cognitive function including memory, language, complex visuospatial processing, and executive function. This validated tool has been helpful to measure the impact of treatments on cognition. Animal models of PD provide a more readily controlled means to assess cellular dysfunction, neurochemical alterations and other neural mechanisms that may contribute to disease pathogenesis in humans.

Exercise is thought to improve overall wellbeing in older adults and benefit cognitive functions of those with neurodegenerative diseases [7]. It has been suggested that exercise may improve the motor manifestations of PD and that restricted use in rats may potentiate neurodegeneration [8]. Specifically, evidence has shown that exercise is beneficial for bradykinesia, postural balance and quality of life in patients with PD [9-12]. The extent to which exercise specifically impacts cognition in PD, and how, is unclear. A non-systematic review from 2011 suggested that vigorous exercise may have a neuroprotective effect in PD [13]. A more recent systematic review similarly showed that non-pharmacological interventions improve cognition in PD. However, this review included only those studies published before December 2011 and used limited search terms related to cognition, resulting in the review of only four clinical studies [14]. A subsequent analysis of the literature was needed to include recent clinical studies and to incorporate animal-based research that might help identify potential mechanisms in humans. Therefore, this systematic review was conducted to evaluate all original research reports that assessed exercise interventions in human PD or in animal models of PD, with a primary or secondary outcome to examine cognitive function. To provide the most comprehensive overview of the literature, non-randomized, pre-post and cohort trials were included in addition to randomized controlled trials. The combination of these findings should be used to further guide clinical practice and neurorehabilitation exercise programs toward treating cognitive deficits in PD.

## Methods

### Systematic review protocol

This systematic review was conducted according to the Preferred Reporting Items for Systematic reviews and Meta-Analyses (PRISMA) statement guidelines [15]. The PRISMA statement includes a 27-item checklist (Additional file 1) and standardized instructions for conducting a systematic review. The complete search methodology, information sources and results for this review are described within this report and Appendix 1.

### Eligibility criteria for study characteristics

The participants included healthy human subjects, subjects with PD and animals with experimental PD. Studies were included if their primary intervention was exercise and their primary or secondary outcomes were to assess either behavioral or neurobiological markers of cognitive function. All articles were included where the authors treated the outcome measure as a test of cognition. In some cases, the measure was a surrogate (e.g., biomarker associated with cognitive function), was a sub-score of cognition from a larger scale, or was influenced by motor capacity. An exercise intervention was defined as any purposeful increase in the subject’s physical activity through a single bout of exercise or prolonged exercise over the course of a structured or unstructured program. Cohort and experimental study designs were included, whereas case series, case–control, cross-sectional and descriptive studies were excluded. Original research articles were included from 1966 through October 2013. Studies were considered if they were written in the English language and either published or “in press”.

## Results

### Study selection and synthesis

**Figure 1:**
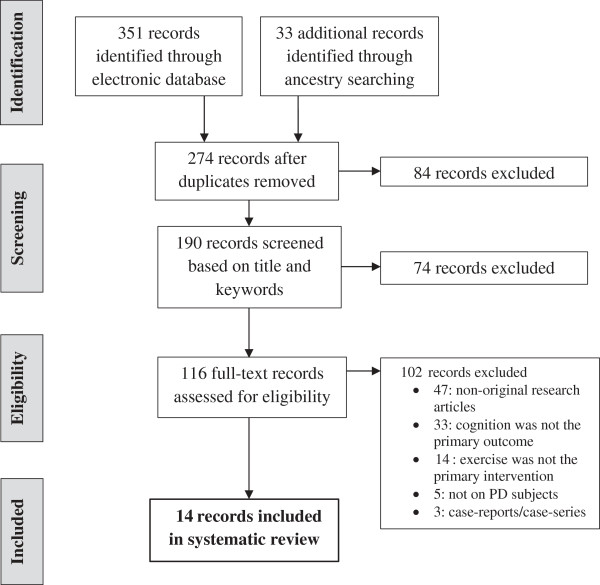
PRISMA Flow Diagram of Study Selection.

There were 14 records included in this analysis (Figure 1). Thirteen records were found through searching databases and one record [16] was found through searching the references of articles identified for inclusion in the analysis. The records comprised six pre-clinical animal studies (all on rodents) and eight clinical studies in humans. Of the six pre-clinical studies, all were randomized controlled studies. Two studies examined the effects of exercise on unspecified aspects of cognition [17,18], and four studies examined the effects of exercise specifically on learning and memory [16,19-21]. Of the eight clinical studies, four studies examined the effects of exercise on unspecified aspects of cognition [22-25], and four studies examined the effects of exercise specifically on tasks of executive function [26-29]. The clinical studies included five randomized controlled trials, one controlled trial and two pre-post trials. A quantitative comparison or meta-analysis could not be performed for either the pre-clinical or clinical studies because there were only a small number of reports identified, and when compiled together they had heterogeneous patient populations, exercise interventions and outcome measures.

### Study characteristics and results for pre-clinical studies

All six pre-clinical studies were randomized controlled trials. Three different toxins were used to generate basal ganglia lesions and develop models of PD in rodents. Three studies used 1-methyl-4-phenyl-1,2,3,6-tetrahydropyridine (MPTP) in mice [16,17,20], two studies used 6-hydroxydopamine (6-OHDA) in rats [18,30], and one study used reserpine in rats [19], resulting in reversible monoamine depletion. The timing of toxin administration varied relative to the onset of the exercise program. Four studies tested the effect of exercise that started following the lesion [16-18,30]. The interventions were five days per week for 30 days starting within a week after administration of either MPTP or 6-OHDA. One study tested the effects of exercise for one week before, five weeks during, and for 8–12 weeks following chronic MPTP administration [20]. The study that used reserpine tested exercise five days per week for 30 days prior to administration of the toxin in order to assess how exercise may prevent cognitive impairment in PD [19].

Four studies looked at forced exercise on a treadmill compared to no exercise [16,18,20,30]. Three studies showed potential neurobiological correlates of observed behavioral changes that the authors related to cognition, including findings from one study that rats forced to exercise had better behavioral recovery on tests of motor function (i.e., cylinder and amphetamine-induced rotational tests), and better preservation of tyrosine hydroxylase immunoreactivity in both striatum and substantia nigra [18]. The authors also found that exercise increased the migration of BrdU and doublecortin-positive cells as well as increased brain-derived neurotrophic factor (BDNF) and glial cell line-derived neurotrophic factor (GDNF) in the striatum on the side of the lesion. In another study, exercise resulted in enhanced duration and velocity of running behavior, indicating that rats had learned to maintain a forward position on a treadmill [16]. Exercise in these MPTP- and saline-injected mice resulted in significant down-regulation of striatal dopamine transporter protein (DAT) as well as increased D_2_ (but not D_1_) receptor mRNA expression. Exercise attenuated the increase in striatal glutamate nerve terminal labeling following MPTP, but there was no change in glutamate immunolabeling in CA1 in the hippocampus. In both of these studies exercise improved behavioral markers that were interpreted by the authors as indicative of enhanced cognitive function. It should be noted that these measures rely heavily on motor capacity and are typically associated more with motor than with cognitive function. The third study that showed neurobiological changes associated with corresponding behavioral changes tested effects of aerobic swimming in mice. The rodents showed improved long-term memory on a test of object recognition following exercise and had attenuation of impairments from exposure to 6-OHDA, including decreased pro-inflammatory cytokines, improved markers of oxidative stress and increased DA transmission [30]. The last study that looked at forced exercise on a treadmill compared to no exercise conducted in a chronic model of PD found that endurance exercise improved only motor function related to gait ambulation and balance, with no improvement in cognitive measures [20]. This study was also the only one of these four where exercise did not improve a neurobiological outcome following toxin administration, including no raise in striatal DA and no reversed loss of tyrosine-hydroxylase fibers in the substantia nigra pars compacta.

Two studies (one with MPTP and one with reserpine) looked at the effects of voluntary exercise (wheel running), compared to forced exercise on a treadmill or no exercise [17,19]. In one study, exercise was introduced prior to the administration of reserpine [19], while in the other [17], exercise was not initiated until after the MPTP lesion. Both studies found that either form of exercise improved behavior underlying cognitive capacity in a PD-like model. Interestingly, only forced exercise, following the lesion, improved a test of motor learning (transfer of treadmill performance to Rotarod). The authors suggested this finding reflects learning as the animals had presumably transferred skill from the treadmill to the Rotarod task [17]. The rodents forced to exercise on the treadmill showed a greater improvement on the Rotarod test, even though rodents on the wheel willingly spent more time exercising than those forced to run on the treadmill. Both voluntary and forced exercise had anxiolytic effects as assessed using the elevated plus maze, which the authors linked to cognition and memory, but neither type of exercise had any effect on depressive behavior as assessed by sucrose preference and tail suspension. These improvements were not associated with changes in the striatal DA or amygdalar serotonin (5HT) levels following the exercise intervention, as compared to saline-treated sedentary controls [17]. However, both MPTP- and saline-treated mice had a similar relative increase in striatal DA following forced or voluntary exercise compared to saline-treated sedentary controls. Forced exercise also increased 5HT in the nucleus accumbens in the MPTP-treated mice compared to saline controls. In the second study, when either forced or voluntary exercise was introduced prior to reserpine administration, both exercise paradigms resulted in improved motor learning on the Rotarod and open-field tasks (tests of exploratory activity), as well as improved social memory [19]. Social memory improved with a low dose or reserpine which, unlike the high dose of the toxin, did not affect the animals’ motor function. Biomarkers were not assessed in this study.

Further details of study characteristics and results for pre-clinical studies are summarized in Tables 1 and 2.

**Table 1:** Study characteristics of pre-clinical studies on rodent models of Parkinson’s disease

**Authors**	**Study title**	**Subjects**	**Intervention**
Goes et al., 2013 [21]	Neuroprotective effects of swimming training in a mouse model of Parkinson’s disease induced by 6-hydroxydopamine	• 2 groups (n = 20 each): 6-OHDA, saline	• 20–60 min/day, 5 days/week for 4 weeks
		• 2 treatment cohorts (n = 10 each): swimming training, no exercise	
			• Starting 4 days after toxin administration
Gorton et al., 2010 [17]	Exercise effects on motor and affective behavior and catecholamine neurochemistry in the MPTP-lesioned mouse	• 2 groups (n = 24 each): MPTP, saline	• Up to 1 hr/day, 5 days/week for 4 weeks
		• 3 treatment cohorts (n = 8/group): forced exercise, voluntary exercise, no exercise	
			• Starting 5 days after toxin administration
Tajiri et al., 2010 [18]	Exercise exerts neuroprotective effects on Parkinson's disease model of rats	• 1 group (n = 60): 6-OHDA	• 30 min/day, 5 days/week for 4 weeks
		• 2 treatment cohorts (n = 30 each): forced exercise, no exercise	
			• Starting 1 day after toxin administration
Aguiar et al., 2009 [19]	Physical exercise improves motor and short-term social memory deficits in reserpinized rats	• 4 groups (n = 24 each): high/low dose reserpine or high/low dose saline	• 20–25 min/day, 5 days/week for 4 weeks
			• Starting 4 weeks before toxin administration
		• 3 treatment cohorts^±^ (n = 8/group):forced exercise, voluntary exercise, no exercise	
Pothakos et al., 2009 [20]	Restorative effect of endurance exercise on behavioral deficits in the chronic mouse model of Parkinson's disease with severe neurodegeneration	• 2 groups (n = 29 each): probenecid/MPTP (model of chronic PD), probenecid only	• 40 min/day, 5 days/week for 8–12 weeks
			• Starting 1 week before, 5 weeks during, 8–12 weeks after toxin administration
		• 2 treatment cohorts (n = 5-10/group): forced endurance exercise, no exercise – for probenicid/MPTP group only	
Fisher et al., 2004 [16]	Exercise-induced behavioral recovery and neuroplasticity in the 1-methyl-4-phenyl-1,2,3,6-tetrahydropyridine- lesioned mouse basal ganglia	• 2 groups (n = 60 each): MPTP, saline	• Up to 2x 30 min/day, 5 days/week for 4 weeks
		• 2 treatment cohorts (n = 20/group): forced exercise, no exercise*	
			• Starting 4 days after toxin administration

^±^Only rats able to maintain a forward position on the treadmill were assigned to the treadmill exercise group.

*Only rats able to maintain a forward position on the treadmill were randomized to either exercise or no exercise cohort.

**Table 2:** Outcomes and risk of bias for pre-clinical studies on rodent models of Parkinson’s disease

**Study**	**Behavioral outcomes**	**Neurobiological outcomes**	**Major sources of risk of bias**
Goes et al., 2013 [21]	*Forced exercise following onset of experimental PD:*	*Changes in the striatum from forced exercise following onset of experimental PD:*	Performance bias: sedentary control animals were not exposed to the swimming training program, the warm water or handled to be dried off following each session.
	• Decreased marker of depression (tail suspension)	• Decreased interleukin 1-beta levels (proinflammatory cytokines)	
	• Improved motor coordination (decreased falls on Rotarod test)	• Attenuated inhibition of glutathione peroxidase activity, decreased glutathione reductase and glutathione S-transferase activity (all markers of oxidative stress)	
	• Improved long-term memory, but not short-term memory in object recognition test		
		• Increased dopamine, homovanillic acid, and 3,4-dihydroxyphenylacetic acid levels	
Gorton et al., 2010 [17]	*Forced and voluntary exercise following onset of experimental PD:*	*Forced and voluntary exercise following onset of experimental PD:*	Performance bias: each animal was only evaluated on one test.
	• Improved motor learning (Rotarod)	• Had no effect on levels of DA in the striatum and serotonin in the amygdala compared to saline controls	
	• Reduced anxiety in elevated plus maze (passive avoidance task, authors linked to cognition/memory)		
		• Forced and voluntary exercise increased DA in the striatum to similar levels following MPTP or saline administration	
	• Had no effect on markers of depression, sucrose preference and tail suspension (MPTP lesion also had no effect)		
		• Forced exercise increased 5HT in the nucleus accumbens in MPTP-treated mice compared to saline controls	
Tajiri et al., 2010 [18]	*Exercise following onset of experimental PD:*	*Exercise following onset of experimental PD:*	Information bias: exercise was started soon (24 hrs) after toxin administration, so the lesion may not represent a complete PD-like model.
	• Improved cylinder test, amphetamine-induced rotational test (authors linked to cognitive-related behavior)	• Preserved nigrostriatal dopamine neurons (increased tyrosine hydroxylase-positive fibers)	
		• Increased migration of new-born neural stem/progenitor cells toward striatum	
		• Up-regulated neurotrophic factors, BDNF and GDNF, in the striatum	
Aguiar et al., 2009 [19]	*Forced and voluntary exercise before onset of experimental PD:*	Neurobiological outcomes not assessed	Information bias: behavioral testing was soon (24 hrs) after the reserpine administration, so the lesion may not represent a complete PD-like model.
	• Improved motor deficits following a high dose of reserpine		
	• Improved short-term social memory (tested through olfactory discrimination), with no deficit on motor or olfactory function from the low dose of reserpine		
Pothakos et al., 2009 [20]	*Endurance exercise before and following onset of experimental chronic PD*:	*Endurance exercise before and after onset of experimental chronic PD:*	Selection biases: there was not a group that received exercise and probenecid. There was also not a control group with only a saline injection. The effects of the control solution, probenecid, on cognition are not known.
	• Reversed balance and gait performance, restored regular movement	• Did not raise striatal DA (n = 6)	
		• Did not reverse loss of tyrosine-hydroxylase fibers in substantia nigra (pars compacta)	
	• Had no effect on learning (cued Morris water maze), amphetamine-stimulated locomotion or motor coordination		
Fisher et al., 2004 [16]	*Exercise following onset of experimental PD:*	*Exercise following onset of experimental PD:*	Information bias: the learning paradigm for behavioral results (learning to stay on the treadmill) relied substantially on motor capacity.
	• Improved velocity and endurance on treadmill	• Had no effect on tyrosine hydroxylase	
	• Sensory feedback not needed over time for behavioral response (i.e., maintaining a forward position on treadmill), authors suggested indicative of learning	• Up-regulated dopamine D_2_ receptor mRNA expression	
		• Down-regulated striatal DAT	
		• Reversed increased nerve terminal glutamate in striatum (as a result of MPTP)	

### Major sources of risk of bias for pre-clinical studies

One of the six pre-clinical animal studies was at risk of selection bias because the investigators did not include a saline-only control group or an exercise and probenecid group, although it was still a randomized controlled trial [20]. Three other pre-clinical studies were at risk of information biases; in one case the cognitive assessment relied on motor capacity [16], in another case the behavioral testing began soon after administration of the toxin, which may have interrupted the lesion process (not measured) and made the model less comparable to PD in human subjects [19] and in the third case the exercise was started soon after the toxin was administered, which may also have interrupted the lesion process [16]. Two studies had performance biases, specifically that each animal was only evaluated on one test [17] and that exercised mice were trained for swimming for two weeks before toxin administration and handled each session, whereas the sedentary animals were not trained or handled [30]. Additionally, the social interaction (i.e., housing environment) the animals experienced was different in each of the six studies, making it difficult to compare results across studies. The number of rodents housed in a cage ranged from one to ten, and one study did not specify the housing environment. Paired housing compared to single housing in rodents has been shown to mediate the effects of MPTP on nigrostriatal degeneration and motor behavior [31] and may have a substantial effect on behavioral and histological outcomes. The risk of bias for each pre-clinical study is summarized in Table 2.

### Study characteristics and results for clinical studies

All eight studies showed that exercise improved a marker of cognition (Table 3). Based on the GRADE ranking system, the quality of the evidence and strength of recommendations for the four studies on executive function were greater (“moderate” and “strong”) than for studies testing unspecified aspects of cognitive function (“low” and “weak”).

**Table 3:** **Quality of the evidence and strength of recommendations for human clinical trials**^**1**^

***Study***	***Can exercise improve a marker of cognitive function?***	***Quality of evidence***^***2***^	***Strength of recommendation***^***3***^
*Studies that specifically measured executive function (n = 4)*			
McKee et al. 2013 [29]	Yes	Moderate	Strong
Cruise et al. 2011 [26]	Yes	Moderate	Strong
Ridgel et al. 2011 [28]	Yes	Moderate	Strong
Tanaka et al. 2009 [27]	Yes	Moderate	Strong
*Studies that measured unspecified aspects of cognition (n = 4)*			
Dos Santos Mendes et al. 2012 [25]	Yes	Low	Weak
Pompeu et al. 2012 [24]	Yes	Low	Weak
Müller et al. 2010 [23]	Yes	Low	Weak
Baatile et al. 2000 [22]	Yes	Low	Weak

^1^Quality of evidence and strength of recommendations based on the Grades of Recommendations, Assessment, Development, and Evaluation (GRADE) ranking system [32,33].

^2^The GRADE system offers four levels of evidence: high, moderate, low and very low.

^3^The GRADE system offers three levels for the strength of a recommendation: strong, weak, or no recommendation.

The sample size for the eight clinical studies varied from six to 60 subjects, with a mean of 15 subjects per group. The PD participants had a mean age between 60 to 70 years and had mild to moderate PD according to the Hoehn and Yahr scale [34], and were compared to age-matched healthy control subjects. All of these studies examined subjects while they were taking their regularly prescribed medication. These studies did not report when subjects took their regular medication and which medications they continued to take during the study.

Each of the four studies that tested the effect of exercise on unspecified aspects of cognition found benefits on general markers of overall function which the authors related to cognition, including the cognition component of PDQ-39, MoCA, memory, reaction time and peg insertion time (requiring visual and spatial cognition, sorting and planning) [22-25]. The evidence from two of the studies is limited as they were pilot trials designed to test feasibility [22,23]. Additionally, the measures of cognition these studies used, the cognition component of PDQ-39, reaction time and peg insertion, are not clear measures of cognitive function. However, the two other studies clearly demonstrated that exercise had an effect on cognitive capacity [24,25]. Of these two trials, both tested the Wii Fit^TM^ program. They showed that after training, PD subjects can retain and transfer learning, depending on the cognitive demands of the game, [25] but Wii Fit^TM^ may not provide additional advantage in comparison to balance exercises without cognitive stimulation or feedback [24].

Across studies, exercise interventions varied significantly in terms of the intensity, mode and duration of the program. One study involved an individualized walking program (*PoleStriding*) using Nordic poles three times per week for eight weeks [22]. Another study assessed outcomes before and after a single bout of high-intensity cycling [23]. The two studies that used the Wii Fit^TM^ program for their exercise intervention included two sessions per week for seven weeks and follow-up after 60 days [24,25]. One of the two studies using Wii Fit^TM^ compared PD subjects to healthy controls [25] and the other study looked at differences between PD subjects participating in the combined Wii Fit^TM^ program with balance-based and cognitive training compared to multimodal global exercises [24].

Each of the four studies that specifically tested executive function showed that exercise improved performance on some measure of executive function, such as tests of abstraction, mental flexibility, spatial working memory, verbal fluency, mental imagery, and cognitive processing speed [26-29]. The tools used to test executive function include the Wisconsin Card Sorting Task, Trail-Making Test A and B, Cambridge Neuropsychological Test Automated Battery, and tests of verbal and semantic fluency. Two studies assessed mood as a potential confounding factor affecting executive function; one study found that exercise improved executive function independent of improvements in mood, attention, disease-specific quality of life or reduced anxiety [27]. Another study found that exercise possibly improved mood, but did not affect quality of life [26]. This lack of impact on quality of life is interesting given that this study did not control for the benefits of social interaction received by the exercise group in comparison to the control group, who were instructed to continue with their normal routine. Both low-intensity passive aerobic cycling [28] as well as moderate-intensity aerobic and anabolic exercise [26,27] were found to improve executive function in PD. The fourth study differed from the other exercise interventions because it assessed 20 sessions of tango classes compared to education over 12 weeks. The subjects were assessed for cognitive function 10–12 weeks following the intervention. Subjects in the tango arm improved on the Brooks Spatial Task, a test of spatial cognition (i.e., mental imagery) [29]. The authors interpreted this as an improvement in executive function.

There was substantial variety in the intensity, mode and duration of the exercise interventions in these four studies. One study included an exercise intervention involving low-intensity passive cycling once per week for four weeks. Another study included moderate-to-high intensity anabolic and aerobic exercise 60 minutes per session twice per week for 12 weeks. The intensity and the work load of the sessions were increased over time. Each session involved a short low-intensity aerobic warm-up, six resistance exercises for both upper and lower body muscle groups, and then 25–30 minutes of aerobic exercise on a stationary bicycle, rowing machine or treadmill. A third study included moderate-intensity multimodal exercise training involving aerobic exercise with the addition of resistance, coordination and balance training 60 minutes per session, three times per week for 24 weeks. The 24-week intervention was divided into six phases and the load was increased after each phase. A session included five components: warm-up, stretching before exercise, exercise, cool-down, and stretching after exercise. The fourth study on tango implemented a standardized structured tango program for 90-minute sessions twice per week for 12 weeks.

Further details of study characteristics and results for clinical studies are summarized in Tables 4 and 5.

**Table 4:** Study characteristics of clinical trials on human Parkinson’s disease

**Authors**	**Study title**	**Subjects**	**Intervention**	**Study design**
*Studies that specifically measured executive function (n = 4)*				
McKee et al. 2013 [29]	The Effects of Adapted Tango on Spatial Cognition and Disease Severity in Parkinson’s Disease	Total n = 33 PD	• Tango or education lessons	Randomized controlled trial
		• n = 15 tango	• Sessions 90 minutes long, 20 sessions over 12 weeks, follow-up after 10–12 weeks	
		• n = 13 control		
Cruise et al. 2011 [26]	Exercise and Parkinson's: benefits for cognition and quality of life	Total n = 28 PD	• Moderate-to-high-intensity anabolic and aerobic exercise or usual care	Single-blind randomized controlled trial
		• n = 15 exercise		
		• n = 13 control	• Sessions 1 hr/day, 2x/week for 12 weeks	
Ridgel et al. 2011 [28]	Changes in executive function after acute bouts of passive cycling in Parkinson's disease	Total n = 19 PD	• Low-intensity passive aerobic exercise (cycling)	Randomized controlled trial, cross-over
			• Sessions 1/week for 4 weeks	
Tanaka et al. 2009 [27]	Benefits of physical exercise on executive functions in older people with Parkinson's disease	Total n = 20 PD	• Moderate-intensity multimodal exercise training (aerobic, resistance, coordination and balance) or usual care	Controlled trial^*^
		• n = 10 exercise		
		• n = 10 control		
			• Sessions 1 hr/day, 3x/week for 24 weeks, intensity increased every 4 weeks	
*Studies that measured unspecified aspects of cognition (n = 4)*				
Dos Santos Mendes et al. 2012 [25]	Motor learning, retention and transfer after virtual-reality-based training in Parkinson's disease - effect of motor and cognitive demands of games: a longitudinal, controlled clinical study	Total n = 27	• Low-intensity Wii Fit^TM^ training, involving motor shifts and cognitive skills	Longitudinal pre-post trial
		• n = 16 PD		
		• n = 11 healthy control	• Sessions 1 hr/day, 2x/week for 7 weeks, follow-up at 60 days	
Pompeu et al. 2012 [24]	Effect of Nintendo Wii^TM^-based motor and cognitive training on activities of daily living in patients with Parkinson's disease: A randomised clinical trial	Total n = 32 PD	• Both groups: low-intensity stretching, strengthening	Single-blind randomized controlled trial
		• n = 16 exercise & Wii		
			• Experimental group: Wii Fit^TM^ -based motor/cognitive training	
		• n = 16 exercise no Wii		
			Control group: balance exercises without feedback or cognitive stimulation	
			• Sessions 1 hr/day, 2x/wk for 7 weeks, follow-up at 60 days	
Müller et al. 2010 [23]	Effect of exercise on reactivity and motor behaviour in patients with Parkinson's disease	Total n = 22 PD	• Single bout of high-intensity endurance aerobic exercise (heart rate-targeted cycling) or rest following L-dopa administration	Randomized controlled feasibility trial, cross-over
			• Randomized order 1 day apart	
Baatile et al. 2000 [22]	Effect of exercise on perceived quality of life of individuals with Parkinson's disease	Total n = 6 PD	• Low-intensity aerobic exercise program with Nordic walking poles (*PoleStriding*)	Nonrandomized feasibility trial, no control
			• Sessions 3x/week for 8 weeks	

*Subjects were assigned into the training group based on previous participation as a control in another study and upon referral by their physician. Baseline characteristics did not differ between the groups.

**Table 5:** Outcomes and risk of bias of clinical trials on human Parkinson’s disease

**Study**	**Behavioral outcomes**	**Major sources of risk of bias**
*Studies that specifically measured executive function (n = 4)*		
McKee et al. 2013 [29]	• Tango improved disease severity (UPDRS-III) and spatial cognition/mental imagery (Brooks Spatial Task) more than education group, maintained gains 10–12 weeks post-intervention	• Detection bias: study was underpowered (n = 23 tango, n = 8 education) to evaluate some main effects within groups, so main effect of time was evaluated
Cruise et al. 2011 [26]	• Exercise improved verbal fluency and spatial working memory on Cambridge Neuropsychological Test Automated Battery	• Selection bias: the control group received usual care, no control for the effect of social interaction with exercise
	• Exercise was of “possible benefit” on semantic fluency and mood	• Information bias: the variable intensity level of the intervention could have affected outcomes
	• Exercise did not benefit spatial or pattern recognition, quality of life, had no negative impact	
Ridgel et al. 2011 [28]	• Time to complete Trail Making Test A & B (tests executive function) decreased after passive cycling	• Selection bias: no control
		• Information bias: the same test pattern was used pre- and post-intervention, although practice effects were attempted to be controlled through pre-test training with the task
	• Performance improved on Trail Making Test B following passive cycling	
Tanaka et al. 2009 [27]	• Exercise improved executive function for “Categories Completed” (i.e., capacity for abstraction) and “Preservative Errors” (i.e., mental flexibility) on the Wisconsin Card Sorting Task	• Selection bias: small sample size, no long-term follow-up, not purely randomized
		•Information bias: no mention of medication administration; only one participant in the group had a heart rate monitor, so the intensity was targeted towards the group and not the individual
	• No interactions for confounding variables: concentrated attention, trait or state anxiety, depression	
*Studies that measured unspecified aspects of cognition (n = 4)*		
Dos Santos Mendes et al. 2012 [25]	• PD showed no deficit in learning or retention on 7/10 games, deficits related to cognitive demands of tasks	• Selection bias: the baseline physical fitness of the subjects was not compared
	• PD had worse performance than healthy individuals on 5 tests	• Performance bias: no PD controls not performing intervention, no control for enjoyment or motivation
	• PD could transfer learning to an untrained motor task at follow-up	
Pompeu et al. 2012 [24]	• Both groups improved UPDRS-II, MoCA and balance, no additional advantage from Wii Fit^TM^	• Information bias: the baseline physical fitness of the subjects was not compared, so potential for differences between groups
	• Improved scores on Wii Fit^TM^ games, maintained at follow-up	
	• No differences in outcomes between groups pre- to post-intervention or in follow-up	
Müller et al. 2010 [23]	• Reaction time increased after rest and decreased after exercise, movement time decreased after exercise	• Selection bias: no PD control group, no healthy controls
		• Information bias: one-day washout period (24 hours) may not have been long enough; pilot trial
	• Number of correct answers decreased after rest	
	• Tapping rate increased after exercise	• Detection bias: unclear how reactivity was measured
	• Peg insertion interval time decreased after exercise (complex movement sequences, visual and spatial cognition, sorting and planning)	
Baatile et al. 2000 [22]	• Improved UPDRS score (only total score significant)	• Selection bias: limited sample size, no control group; pilot trial
	• Improved PDQ-39 score, most improved in cognition component	• Information bias: exercise intensity not standardized

### Major sources of risk of bias for clinical studies

Most of the studies had a selection bias from not adequately standardizing control subjects. More specifically, the subjects’ physical fitness levels and their concomitant medications before and during the study period were not documented and may have impacted their ability to exercise and the ability to compare their potential benefits on cognition. Additionally, information bias resulted from variability with the timing and intensity of the exercise intervention between subjects. Individuals may have received different amounts and types of exercise which could have affected the impact of exercise on cognition.

Of the five randomized controlled clinical trials, four studies included control groups that received the same social interaction as the exercise intervention group. One study [26] included a control group receiving usual care, which did not control for the potential benefits to the subjects’ mood and cognition from increased social interaction through participation in the exercise intervention. Overall, both the pre-clinical and clinical studies showed a trend of selective reporting for only significant outcomes. A risk of bias across studies includes a publication bias, as studies with insignificant or negative findings are less likely to be published. The risk of bias for each clinical study is summarized in Table 5.

## Discussion

### Pre-clinical evidence

The rodent studies identified in this review have suggested potential mechanisms for the benefits of exercise on cognitive improvements in PD, particularly related to learning and memory. The mechanisms include:

1) enhanced availability of DA in projections to the dorsal and/or ventral striatum;

2) enhanced expression of neurotrophic factors BDNF and GDNF, which could promote plasticity for learning and memory; and

3) decreased oxidative stress and/or neuroinflammation in the basal ganglia.

Two of the five studies that assessed biomarkers did not find effects from exercise following toxin administration. However, in one case forced and voluntary exercise similarly elevated striatal DA in both MPTP- and saline-treated groups, although there was no difference compared to sedentary saline-treated controls [17]. The other study used a model of chronic PD where the toxin was administered with probenecid over five weeks; while exercise restored regular motor function, it had no effect on learning [20]. DA levels following the chronic toxin administration were low and potentially more difficult to increase with exercise due to the substantial neurodegeneration.

The obvious caveat to all studies using rodents to assess cognition is that the tasks of cognition, learning and memory require motor function to evaluate their performance. The models used in these studies (i.e., MPTP, 6-OHDA and reserpine) are widely accepted, although none of them recapitulates the indolent and progressive nature of PD [35-40]. It is unknown how the time of onset or intensity of the exercise paradigm initiated during or following the lesion/toxin may affect the severity of parkinsonism. More promising models for the future would include transgenic or knock-in rodents characterized by expression of mutant or overexpression of wild-type α-synuclein. A related model is the injection of synthetic α-synuclein fibrils into the rodent brain. This model shows progressive and selective loss of DA neurons in the substantia nigra pars compacta, as well as cell-to-cell transmission in anatomically connected regions [41]. As cortical Lewy body pathology is a major contributing factor to dementia in PD [42], future research with models of abnormal α-synuclein deposition may translate better into clinical research and practice than models that create basal ganglia lesions.

The potential for exercise to improve cognition by reducing the impact of neuroinflammation is promising. Another study in a rodent model not dependent upon a selective dopaminergic neurotoxin found that forced treadmill and voluntary wheel exercise in rats can also alleviate impairments from brain inflammation on long-term potentiation and spatial learning [43]. The authors injected rats with lipopolysaccharides into the cerebral ventricles to induce brain inflammation. They found that both treadmill and wheel training improved the resulting deterioration in spatial learning. The effects of exercise on learning were attributed to enhanced expression of BDNF, tyrosine kinase B and phosphorylated cyclic AMP response element binding protein in the hippocampus. The impact of the injection on neuroinflammation was not documented. This model results in preferential but not entirely selective nigral DA cell degeneration and the findings may thus provide insight into potential mechanisms of exercise in humans to reduce brain inflammation and improve cognition.

Overall, the results from these studies on rodent models of PD offer promising support for exercise to improve cognition in humans with PD through the promotion of neuronal proliferation, neuroprotection, neurogenesis, and potentially reduction in brain inflammation. These results support recent work that highlights how exercise likely promotes neurorestoration through activation of signaling cascades by neurotrophic factors [44]. Exercise has been shown to affect regulation of DA function, including upregulated DA D_2_ receptor mRNA and down-regulated striatal DAT in rodents [16]. High-intensity exercise also increased DA D_2_ receptor availability in a recent feasibility study by the same group using five human subjects (n = 2 PD exercise, n = 2 PD no exercise, n = 1 healthy control) and positron emission tomography with [^18^F]fallypride [45]. While this small human trial does not assess cognition, these findings encourage more clinical trials based on rodent outcomes in this field. Overall, the evidence from rodent studies in this systematic review cannot be directly applied to mechanisms in humans yet, but they suggest that PD patients would likely experience a meaningful improvement in cognition in response to exercise.

### Clinical evidence

Clinical studies in humans demonstrate that various modalities and intensity levels of exercise can improve cognitive capacity in PD, and especially executive function, although the mechanisms have not yet been determined. Cognitive dysfunction in PD is commonly associated with impaired executive function [46]. However, evaluating research on cognition, and particularly executive function, in PD is challenging because mild cognitive impairment (MCI) in PD has only recently been defined and formal diagnostic criteria are still in development [47]. Selection and interpretation of measures of executive function in PD have been challenging and the clinical implications are not yet fully appreciated [48]. Executive function is generally related to goal-directed behaviors processed by the frontal lobes of the brain. Executive function has been categorized into four components: planning, purposive action, effective performance and volition [49]. It is not known what frequency, intensity, type or timing of exercise might be most effective to improve executive function in PD, but there is evidence in older adults without PD that light aerobic exercise (walking), and not anaerobic exercise (stretching and toning), selectively improves executive functions processed in the frontal and prefrontal areas of the brain [50]. The potential different effects of aerobic compared to anaerobic exercise on cognition in PD have not yet been studied.

Exercise in animal models of PD may induce DA release and enhance DA transmission via up-regulation of DA D_2_ receptors [51,52]. A systematic review on the effects of exercise in the elderly showed that moderate-intensity exercise can effectively increase peripheral BDNF [53]. Serum BDNF crosses the blood–brain barrier so these results may have implications for brain neurotrophin levels [54]. One of the animal studies reviewed in this paper also found that exercise increased BDNF and GDNF in the striatum [18]. The findings from this review support the theory of potential neuroprotective benefits of exercise for human PD. A recent review on the benefit of exercise to improve cognition emphasized the potential neuroprotective effects of vigorous exercise in PD [13]. The authors provided guidelines including vigorous exercise, structured programs for cognitively impaired patients, and therapies that replenish DA to provide the maximum capability and motivation to exercise [13]. In addition to these guidelines, the current systematic review shows that any exercise should be encouraged as it may benefit numerous aspects of patients’ cognitive function and these effects could be transferrable to other tasks. Importantly, the effects of vigorous exercise can last up to 60 days [25,29]. In these studies there was overall a high retention rate for subjects committing to a twice or three times weekly exercise program, suggesting that these interventions could be feasibly implemented as treatment programs. A recent meta-analysis showed that very light to vigorous exercise seems to have a small effect on cognition in the acute phase following exercise, but larger longer-lasting effects are possible with more intense exercise [55].

The benefits of exercise on cognition in PD are comparable to those seen in healthy older adults. A recent review showed that endurance and resistance exercise can improve cognition in healthy seniors [7]. There is less research on the effects of exercise in frail older adults, but recent evidence showed that a three-month physical activity intervention improved physical abilities, executive functions, processing speed and working memory [56]. The effects of exercise on cognition in older adults with MCI are less promising, as a recent meta-analysis showed only limited potential to improve cognition [57]. However, the interpretation is constrained because many of the publications were deemed by the authors of this analysis to be of moderate quality and many of the studies were underpowered. It is possible that exercise may have an impact on dopaminergic signaling that renders it particularly valuable in PD. Whether cognition in PD is improved due to dopaminergic mechanisms of exercise or other mechanisms such as increased neurotrophic factor availability or reduced neuroinflammation remains to be determined.

### Limitations

Limitations at the level of each study include risk of information, performance and/or selection biases (Tables 2 and 5) as well as confounds inherent with the limitations of non-randomized trials. There was overall a lack of reporting of concomitant medications (i.e., PD therapies and antidepressants) as well as the medical condition of the subjects, which could have affected the results. The limitations of this review include potential for incomplete retrieval of information given the search strategy and inclusion criteria. Additionally, it is not known whether there are genetic factors underlying response to exercise in PD.

## Conclusions

Overall, this systematic review found that exercise can improve cognitive function in animal models and human PD. Pre-clinical studies showed exercise results in behavioral and corresponding neurobiological changes in the basal ganglia related to cognition. Specifically, learning and memory improved after exercise in the rodents, although the exact mechanisms remain unclear and merit further research. Pre-clinical studies also showed that any exercise is better than inactivity and that forced exercise has a greater impact than self-paced voluntary exercise. Exercise resulted in structural, neurochemical and molecular changes in rodents, which may be of relevance to the human disorder. The clinical studies showed that various types of exercise, including aerobic, resistance and dance can improve cognitive function, especially executive function in PD patients. However, the best type, amount, mechanisms, and duration of exercise are not yet known. The evidence from clinical studies suggests that a more intensive aerobic exercise program including strength and balance training can promote greater cognitive gains. However, low-intensity exercise and balance-based exercises also showed benefits.

Research on the effects of exercise on cognition in PD is a relatively new area. As outlined in this review, there are several limitations with the current studies in terms of study design and risks of bias. Questions that remain to be addressed include the prescription of exercise, if any, which elicits the most gains as well as the duration of effects. Future research on the effects of exercise on cognition in PD should include a longitudinal randomized controlled clinical trial examining neurobiological mechanisms *in vivo*, including neuroimaging. Understanding the mechanism of benefit from exercise could help us to harness its potential neuroprotective effects. Patients should use these findings as further rationale to increase their daily physical activity. With growing support for exercise to improve not only motor symptoms, but also cognitive impairments in PD, health care providers and policy makers should recommend exercise as part of routine management and neurorehabilitation for PD.

## Appendix

### Appendix 1: Methodology on information sources, study search and collection

The following electronic databases were searched for articles: PubMed, Web of Knowledge, and EBM Reviews (OvidSP). The primary search parameter for each database used the keywords, “Parkinson’s disease” AND “exercise” AND “cognition” with the Boolean operator “AND”. Additional keywords related to cognition were also searched by replacing “cognition” in the primary keyword phrase one at a time with each of “dementia”, “Alzheimer”, “memory”, “executive function”, and “impulse”. Additional studies were identified by ancestry searches of the articles yielded from the electronic search. No limits were provided for any of the database searches. Study author DM performed all aspects of the search, screen and identification of eligible studies (Figure 1). All studies identified through the information sources were compiled on the citation manager, EndNote. The titles of each study were then screened to identify PD participants, including human subjects or PD-like animal models. The abstracts of each remaining study were then searched manually for the eligibility criteria. The full text was searched for articles deemed to meet the eligibility criteria based on their title and abstract. All data were extracted by the study author DM in their existing form from the articles. The collected data and risk of bias [32] was used to assess the clinical studies for their quality of the evidence and determine the strength of the recommendations (Table 3) based on the Grades of Recommendations, Assessment, Development, and Evaluation (GRADE) ranking system [33]. The clinical studies were assigned the same GRADE rankings by two independent raters (DM and MS).

## Abbreviations

MPTP: 1-methyl-4-phenyl-1,2,3,6-tetrahydropyridine; 5HT: Serotonin; 6-OHDA: 6-hydroxydopamine; BDNF: Brain-derived neurotrophic factor; BDNF: Bromodeoxyuridine; DA: Dopamine; DAT: Dopamine transporter; EF: Executive function; GDNF: Glial cell-derived neurotrophic factor; L-dopa: Levodopa; LTP: Long-term potentiation; MCI: Mild cognitive impairment; MoCA: Montreal Cognitive Assessment; MPTP: 1-methyl-4-phenyl-1,2,3,6,-tetrahydropyridine; ORP: Overall Rotarod performance; PDQ-39: Parkinson’s Disease Questionnaire; PD: Parkinson’s disease; p-CREB: Phosphorylated cyclic AMP response binding protein; 5HT: Serotonin; PDQ-39-SI: Parkinson’s Disease Questionnaire-39-Single Index; TH: Tyrosine hydroxylase; TMT: Trail-Making Test; Trk-B: Tyrosine kinase B; UPDRS: Unified Parkinson’s Disease Rating Scale.

## Competing interests

The authors declare that they have no competing interests.

## Authors’ contributions

DM conducted all aspects of the literature search and primary preparation of this manuscript. All authors critically revised drafts of this manuscript, and read and approved the final manuscript.

## Authors’ information

Danielle K. Murray is a graduate student in the Graduate Program in Neuroscience at the University of British Columbia.

Matthew A. Sacheli is a graduate student in the Graduate Program in Neuroscience at the University of British Columbia.

Janice J. Eng is a Professor in the Department of Physical Therapy at the University of British Columbia.

A. Jon Stoessl is a Canada Research Chair in Parkinson’s disease, Director of the Pacific Parkinson’s Research Centre and Professor and Head of Neurology at the University of British Columbia.

## Supplementary Material

Additional file 1 PRISMA Guidelines Checklist. Click here for file
